# Neural Correlates of Attentional Modulation of Prepulse Inhibition

**DOI:** 10.3389/fnhum.2021.649566

**Published:** 2021-06-21

**Authors:** Ming Lei, Yu Ding, Qingxin Meng

**Affiliations:** ^1^Laboratory of Artificial Intelligence and Cognition, School of Tourism Sciences, Beijing International Studies University, Beijing, China; ^2^Division of Sports Science and Physical Education, Tsinghua University, Beijing, China; ^3^Collaborative Innovation Center for Brain Disorders, Beijing Institute for Brain Disorders, Capital Medical University, Beijing, China

**Keywords:** prepulse inhibition, attention, event-related potentials, N1, sensory gating

## Abstract

Prepulse inhibition (PPI) refers to the suppression of the startle reflex when the intense startling stimulus is shortly (20–500 ms) preceded by a weak non-startling stimulus (prepulse). Although the main neural correlates of PPI lie in the brainstem, previous research has revealed that PPI can be top-down modulated by attention. However, in the previous attend-to-prepulse PPI paradigm, only continuous prepulse but not discrete prepulse (20 ms) could elicit attentional modulation of PPI. Also, the relationship between the attentional enhancement of PPI and the changes in early cortical representations of prepulse signals is unclear. This study develops a novel attend-to-prepulse PPI task, when the discrete prepulse is set at 150 ms at a lead interval of 270 ms, and reveals that the PPI with attended prepulse is larger than the PPI with ignored prepulse. In addition, the early cortical representations (N1/P2 complex) of the prepulse show dissociation between the attended and ignored prepulse. N1 component is enhanced by directed attention, and the attentional increase of the N1 component is positively correlated with the attentional enhancement of PPI, whereas the P2 component is not affected by attentional modulation. Thus, directed attention to the prepulse can enhance both PPI and the early cortical representation of the prepulse signal (N1).

## Introduction

The startle reflex is the whole-body reflexive response to sudden and intense sensory stimuli, such as an intense startling broadband noise (pulse) (Landis and Hunt, 1939). Prepulse inhibition (PPI) refers to the reduction in the magnitude of the startle reflex produced by a weak non-startling stimulus (acoustic, visual, or tactile prepulse) presented shortly (20–500 ms) before the startle stimulus (acoustic or tactile pulse) (Hoffman and Ison, 1980). The “protection-of-processing” theory proposed by Graham suggests that the startle response interrupts information processing, and PPI protects that processing from interruption. Receiving a prepulse simultaneously triggers the information processing for the prepulse and the gating mechanism dampening the disrupting effect of the startle response (Graham, 1975; Blumenthal et al., 2015). Since the consequences of PPI include the reduction of behavioral responses to startle stimuli by regulating the motor/premotor system, PPI has been generally considered as an operational measure of sensorimotor gating for both humans and animals (Lei et al., 2018; Ding et al., 2020).

Although the basic neural circuitry mediating PPI mainly lies in the brainstem and PPI is thought to be automatic, PPI can be top-down modulated by higher-order cognitive processes, such as attention and emotion (Filion et al., 1993; Du et al., 2011; Ding et al., 2020). In humans, attention can modulate the magnitude of PPI (Dawson et al., 1993; Filion et al., 1993; Hazlett et al., 2003, 2007; Poje and Filion, 2021). In the attention-to-prepulse PPI paradigm developed by Filion et al. (1993) and replicated many times (Hazlett et al., 2003, 2007; Poje and Filion, 2021), subjects are presented with an intermixed series of high and low pitch tones and are instructed to count the number of longer-than-usual occurrences (7 s rather than 5 s) of one particular pitch (Filion et al., 1993). The rationale for this paradigm is that the task requires subjects to discriminate between to-be-attended and to-be-ignored pitches and then sustain selective attention only to the to-be-attended pitch in order to determine its length (Filion et al., 1993). In this PPI paradigm, PPI is greater when the prepulse is attended than when ignored.

However, in this study, the attentional modulation of PPI can only be elicited by continuous prepulse (prepulse that temporally offset at or beyond the onset of the startle stimulus), but not discrete prepulse (prepulse that temporally offset prior to startle stimulus onset) at a lead interval of 120 ms (Poje and Filion, 2021). One possible explanation is that when the prepulse is discrete and the duration is 20 ms, participants cannot discriminate tone frequency within 20 ms for the target and non-target tones to have different effects on PPI. Previous studies using longer discrete prepulse (50–150 ms) have shown that PPI can be modulated by fear conditioning and perceptual separation in animals and human models (Li et al., 2009; Du et al., 2011; Lei et al., 2014, 2018; Wu et al., 2019). Yet, it is unclear whether long discrete prepulse could elicit an attentional effect on PPI. Thus, in the present study, we first develop a novel attention-to-prepulse PPI paradigm to investigate whether attended prepulse could induce larger PPI than ignored prepulse, when the prepulse is discrete and the prepulse duration is set at 150 ms.

In addition, PPI is modified by attentional processes depending on the lead interval used (Filion et al., 1993; Heekeren et al., 2004; Li et al., 2009; Lei et al., 2018). The lead interval refers to the time from prepulse onset to startle stimulus onset and is considered a very important parameter in determining PPI, especially if we are trying to understand the flow of information through neural pathways. In Filion et al. protocol (1993), attentional modulation of PPI occurs at 120 ms but not 60 or 240 ms lead interval. However, long lead intervals have also been approved to be efficient in eliciting modified PPI in other studies. For example, PPI modulated by fear conditioning occurs at the lead interval of 100 ms (Du et al., 2011; Lei et al., 2014). PPI was modulated by perceptual separation at the lead interval of 210 and 270 ms (Lei et al., 2018; Wu et al., 2019), and PPI with different modalities for the prepulse and the startle stimulus was modulated by attention at a lead interval of 300 ms (Bradley et al., 1993). Thus, it would be of interest to investigate whether PPI induced by discrete prepulse can be modulated by attention with long lead intervals. As such, we will adapt a prepulse-counting task that involves active attentional processing of prepulse stimuli and examine whether PPI can be modulated by directed attention, with discrete prepulse at a lead interval of 270 ms.

In addition, there is evidence from animal studies that PPI is modulated by cortico-striato-thalamicpallido-pontine circuitry (for review, refer to Koch and Schnitzler, 1997; Swerdlow et al., 2001). Functional magnetic resonance imaging studies showed that greater activation existed in the dorsolateral pre-frontal cortex, striatum, and the thalamic mediodorsal nucleus during attended than ignored PPI conditions (Hazlett et al., 2008). However, there is a lack of electroencephalogram (EEG) study investigating the cortical neural pathways underlying attentional modulation of PPI. One recent study has revealed that PPI is enhanced when the perceptual separation between the prepulse and noise background is introduced (Lei et al., 2018). Primarily, perceptual separation could enhance the early cortical responses (N1/P2 complex) to the prepulse, and the increase of the N1 component, which is induced by perceptual separation, is positively correlated with the PPI increase, whereas the P2 component increase is not correlated with the PPI increase. It is well-known that the N1/P2 ERP complex, a group of ERP complex of the early cortical auditory potentials, are reliably elicited by various acoustic stimuli, such as pure tones, single syllables, and noise burst (Zhang et al., 2014, 2019). In most cases, the N1/P2 components are elicited at the same time, but these two components are considered to originate from different brain regions, and function differently (Woods et al., 1993). The N1 component may be produced in the temporal auditory cortical fields, which include both the Heschl's gyrus as well as the superior temporal polysensory area (STP), while the P2 component may reveal the acoustically driven outputs from the mesencephalic reticular activating system, which reflects multiple sensory inputs (Crowley and Colrain, 2004). The work by Annic et al. (2014) demonstrated that stimulus-driven attention to the prepulse can affect N1, while goal-directed attention to the prepulse can affect P2. Zhang et al. (2019) have reported that a target syllable /bi/ induces steady N1/P2 complex, and N1/P2 was enhanced by spatial attention to the target syllable. However, the P2 wave but not the N1 wave was affected by perceptual separation when the target sound was attended. Thus, the second purpose of this study is to investigate whether the two ERP components (N1 and P2) of the prepulse stimulus are involved in the novel attention-to-prepulse PPI in different manners.

## Materials and Methods

### Participants

Eighteen healthy adults (10 males and 8 females, mean age = 21.88 ± 1.87 years) participated in this study. All the participants were right-handed native Chinese speakers with normal (audiometric thresholds <25 dB HL between 250 and 8,000 Hz) and bilaterally balanced hearing (interaural threshold differences at each of the frequencies did not exceed 10 dB). The participants were paid a modest stipend ($15.00) for their participation. One participant had to be excluded because of a noisy electromyogram (EMG) baseline and >50% rejected trials in the PPI test. Seventeen participants were included in the final data analysis. We have calculated a power analysis with the G^*^power software. The alpha is set at 0.05, the total sample size is 17, the effect size is set at 0.7, then the calculated statistical power is 0.86, which is considered as acceptable power (Faul et al., 2007, 2009).

This study was conducted according to the principles expressed in the Declaration of Helsinki. The procedures of this study were approved by the Ethics Committee of the Laboratory of Artificial Intelligence and Cognition at Beijing International Studies University. All participants gave written informed consent before they participated in this study.

### Apparatus and Stimuli

In the PPI experiment, each participant was seated in a recliner chair in a sound-attenuated room. A ground electrode was positioned behind the right ear, and two 4 mm Ag/AgCl electrodes were put lateral and below to the right eye over the orbicularis oculi. Electrode resistances were <5 kΩ. The eyeblink component of the acoustic startle reflex was collected with a human EMG startle reflex system (EMG XEYE human startle reflex, Tianminghongyuan Instruments, Beijing, China). Electromyogram activities were band-pass filtered (10,500 Hz) and amplified by 40,000. The electrical voltage was collected, and then sampled at a frequency of 1,000 Hz for 450 ms (150 ms before and 300 ms after the startling stimulus onset). The peak amplitude of the startle response within the time window of 20–300 ms after the onset of the startle stimulus for a single trial was digitized and measured.

The prepulse were 150 ms 65 dB SPL(A) narrowband noises including 15 ms rise time at the beginning and 15 ms fall time at the end of the prepulse, with a center frequency of 200 and 500 Hz. Gaussian wideband noise signals were synthesized using the “randn()” function in the MATLAB function library (the Math Works Inc., Natick, MA, USA) at the sampling rate of 16 kHz with 16-bit resolution, which was then band-pass filtered with a bandwidth of one-third octave and a center frequency of 200 or 500 Hz. The startle-eliciting stimulus was a 104 dB SPL(A) white noise burst of 40 ms in duration with a near-instantaneous (<1 ms) rise-fall time. The background noise was continuous 60 dB white noise. All the acoustic signals were calibrated by a sound-level meter (AUDit and System 824, Larson Davis, USA). They were delivered from a notebook computer sound card (ATI SB450 AC97) and presented by headphones (HD 265 linear, SENNHEISER, 10–25,000 Hz, Germany).

In the scalp EEG recording experiment, acoustic signals (the 200-Hz and 500-Hz narrowband noises used as prepulse in Experiment 1) were transferred using a Creative Extigy sound blaster and presented to participants by two tube ear inserts (ER-3, Etymotic Research, Elk Grove Village, IL) in EEG recordings. EEG recordings were conducted in the sound-attenuating booth (EMI Shielded Audiometric Examination Acoustic Suite) that was equipped with a 64-channel Brain Products system (Brain Products GmbH, Germany).

### Testing Procedures

Each participant underwent two experiments. In Experiment 1, the PPI induced by attended and ignored prepulse was tested. In Experiment 2, the EEG evoked by attended and ignored prepulse was recorded. Experiment 1 was conducted first, followed by Experiment 2 for each participant. The time interval between the two experiments was about 40 min.

**Experiment 1:** In the PPI experiment, the participants were instructed to listen closely to a series of high (center frequency = 500 Hz) and low narrowband noises (center frequency = 200 Hz) presented through headphones. Half of them were instructed to count silently the number of high-pitched target noises (500 Hz), and to ignore the low-pitched noises (200 Hz). Half of them were instructed to count silently the number of low-pitched target noises (200 Hz), and to ignore the high-pitched noises (500 Hz). Participants were informed that a brief loud noise (the startle stimulus) might be presented during the test but that was unrelated to the test.

After the instruction, the participants were given 3 presentations of the loud noise burst alone to calibrate eyeblink amplitude. Two examples of the 500 Hz narrowband noise and two examples of the 200 Hz narrowband noise were then presented. Half of them need to attend to the 500 Hz narrowband noise and half of them need to attend to the 200 Hz narrowband noise. All participants confirmed that they could discriminate between the high and low narrowband noises.

At the beginning of PPI testing, a 3 min adaptation period was introduced with a broadband background noise (60 dB SPL). During the testing, in addition to the prepulse (500 and 200 Hz narrowband noises, 150 ms, 65 dB SPL), a masker (the broadband noise, 0–10 kHz, 60 dB SPL) was continuously delivered from the two headphones. In a prepulse with startle testing trial, the startling white-noise burst (40 ms, 104 dB SPL) started 120 ms after the offset of the prepulse, and the lead interval (the time between the prepulse onset and the startle onset) was 270 ms. The next testing trial started about 15 s later (varying from 10 to 20 s). In total, three types of trials were used: (1) 16 500 Hz narrowband noises; (2) 16 200 Hz narrowband noises; and (3) 8 startling-pulse-alone trials. For each participant, all the 40 trials were presented in a pseudo-random order.

**Experiment 2:** In the EEG experiment, EEG responses to the prepulse stimulus were collected. Note that in Experiment 2, only the prepulse and the noise masker were presented (the startling sound was not presented). During the EEG recording, both the target sound (the prepulse stimulus as used in Experiment 1) and the noise masker were presented from the two eartubes. There were 440 trials in total, and the duration for each trial was 2,000 ms. Among the 440 trials, 200 trials presented both the 200 Hz narrowband noise and the masker, 200 trials presented both the 500 Hz narrowband noise and the masker, and the remaining 40 trials presented the masker only. The overall 440 trials were divided into 2 blocks. In a block, 200 and 500 Hz narrowband noise trials were presented 100 times and 20 trails were masker noise without narrowband noise. A single trial started with the masker, and then the prepulse (target stimulus) was presented within an 800–1,000 ms temporal window after the masker onset. The high-pitch (low-pitch) participants in Experiment 1 were also instructed to attend to the 500 Hz (200-Hz) narrowband noise in Experiment 2. Participants were asked to press a button if they heard the target after the offset of the masker. In order to limit eye movements, a cross in the center of the monitor screen was presented to participants in front of them for watching. The trial interval was 2,000 ms.

### Data Recording and Analyses

In the PPI experiment, startle eyeblink responses were recorded with the startle equipment as EMG activity. Each trial was visually inspected for voluntary and spontaneous blinks. The value of “amplitude to startling sound alone” was calculated as an average of the peak values of the startle-eyeblink EMG among the startle-alone trails. The magnitude of PPI was calculated, in general, with the following formula:

PPI = (amplitude to startling sound alone – amplitude to startling sound preceded by prepulse)/(amplitude to startling sound alone).

In the EEG experiment, EEG signals were recorded with the Vision Acquire 4.3 software (Brain Products GmbH, Germany). All electrodes were referenced to the electrode located on the head center during recordings. The online bandpass was set at 0.05–40 Hz, and the sampling rate was set at 1,000 Hz. Electrodes located superiorly and inferiorly to the left eye and at the outer canthi of the two eyes were used to check for eye movements and eye blinks. Preprocessing and analyses were conducted using MATLAB (MathWorks) and ERPlab toolbox (Luck, 2014). EEG was re-referenced offline to an average of left and right mastoids and bandpass filtered (0.1–30 Hz, 48 dB/octave). The signals were then cut into epochs from −100 to 500 ms relative to the onset of the prepulse target. All epochs were corrected relative to the baseline from −100 to 0 ms. Artifacts that were excessive peak-to-peak deflection (±100 μV) were removed before averaging. ERPs were averaged separately for each combination of electrode site and stimulation condition for each participant. The ground averaged ERPs evoked by the attended prepulse and ignored prepulse were analyzed across participants. We investigated the latencies and voltages of the N1 (the largest negative potential 100–210 ms after the target onset) and P2 components (the largest positive potential 210–350 ms after the target onset). These temporal windows were consistent with previous studies (Zhang et al., 2014; Lei et al., 2018). In addition, the averaged responses at the Cz electrode were statistically analyzed.

For the PPI experiment and EEG experiment, data obtained from the 200 and 500-Hz narrowband noises were pooled together because results obtained from the two noises exhibited similar patterns. The null-hypothesis rejection level was set at 0.05. Repeated-measures ANOVAs followed by Bonferroni pairwise comparisons and paired *t*-tests were performed using SPSS 22.0 software.

## Results

### Results of PPI

In Experiment 1, participants were asked to report the number of the target prepulse in order to instruct the attention of participants to the attended prepulse. All the 17 participants reported 16 ± 2 trials, demonstrating that attention of participants to the attended stimuli was well-maintained.

**Figure 1:**
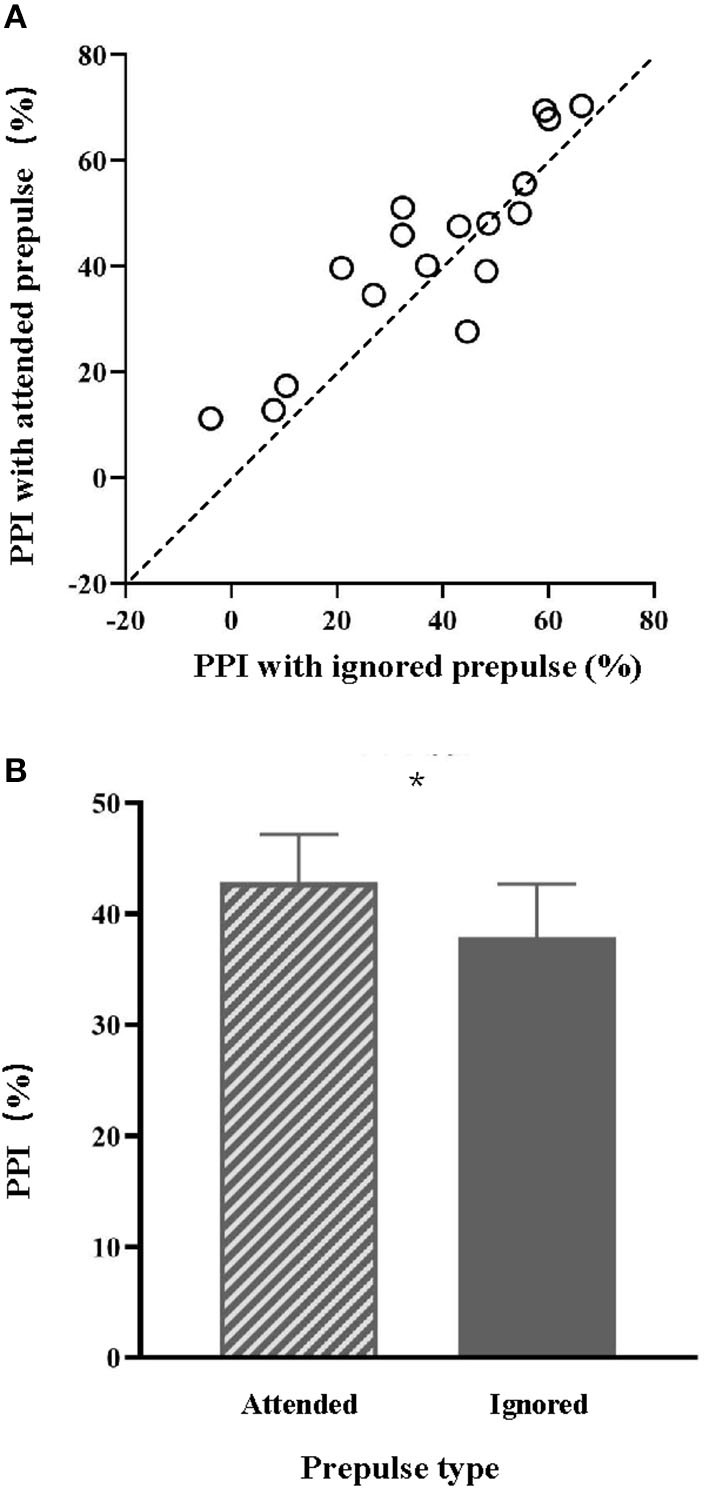
**(A)** The PPI values with the attended prepulse or with the ignored prepulse for the 17 individuals in Experiment 1. **(B)** Comparisons of the mean PPI magnitude between the attended prepulse condition and the ignored prepulse condition. *p < 0.05.

The PPI values of the 17 individual participants under the attended-prepulse condition (values alone the ordinate) and those under the ignored-prepulse condition (values along the abscissa) are shown in Figure 1A. PPI values under the attended-prepulse condition were larger than the PPI values under the ignored-prepulse condition (Figures 1A,B). Paired *t*-test showed that PPI induced by attended prepulse (*M* = 42.84 and *SD* = 4.42) was significantly larger than the PPI induced by ignored prepulse (*M* = 37.91 and *SD* = 4.89) [*t* (16) = 2.15 and *p* = 0.047].

### Amplitudes of ERPs to the Prepulse Stimuli

**Figure 2:**
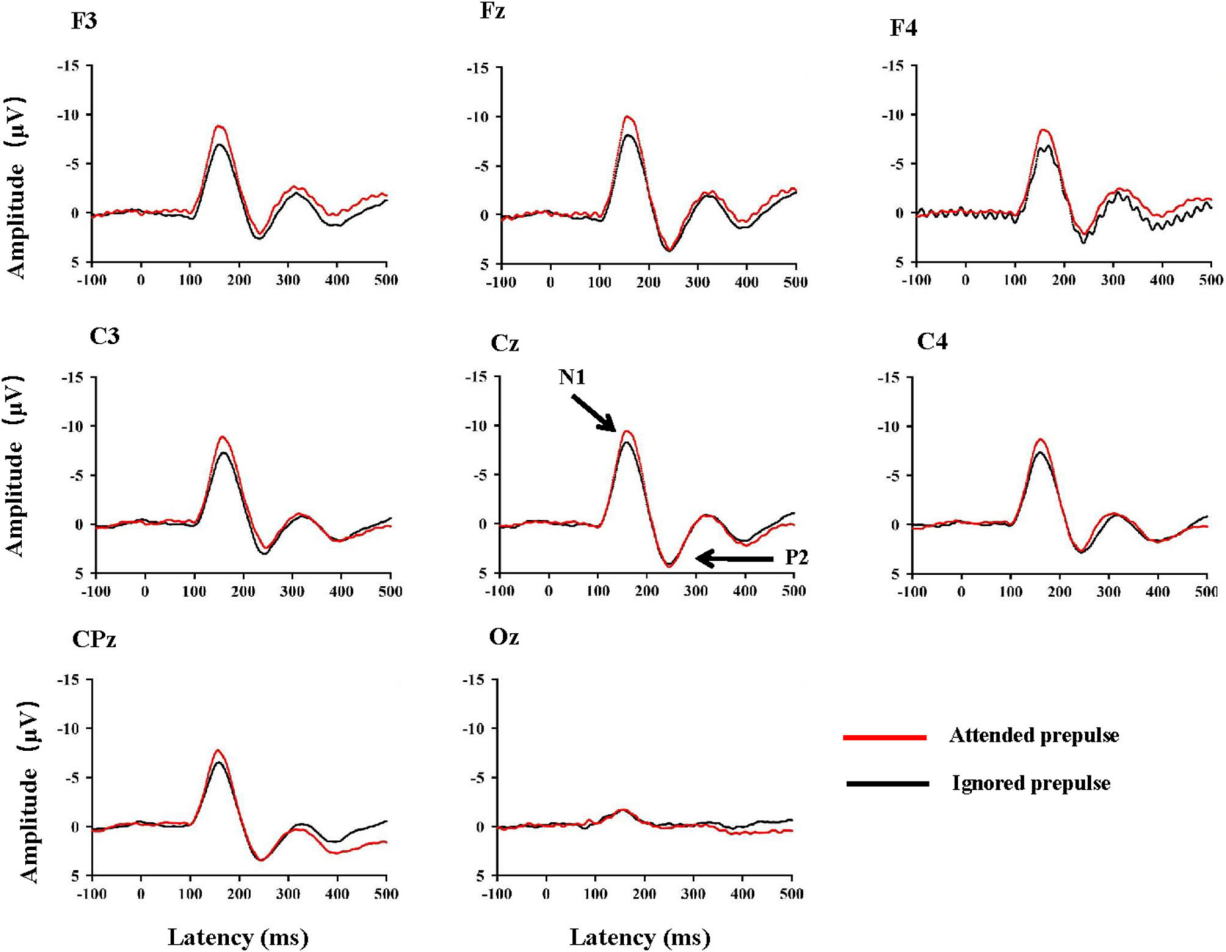
Group-mean ERP waveforms at typical electrode sites F3, Fz, F4, C3, Cz, C4, CPz, and Oz in the attended prepulse condition and the ignored prepulse condition. Note that for the electrode sites surrounding the site Cz, the average amplitude to the attended prepulse was larger than that of the ignored prepulse.

Figure 2 shows the ERP waveforms at typical electrodes, including F3, Fz, F4, C3, Cz, C4, CPz, and Oz under the two conditions. The N1/P2 complex was salient at the fronto-central electrode sites and did not exhibit obvious differences between the left and right hemispheres. As shown in Figure 2, the attended stimulus evoked a larger N1 amplitude than the ignored stimulus. On the contrary, the P2 amplitude was not affected by the attentional manipulation. Since the N1/P2 complex at the center site (Cz) was the most salient (also refer to Zhang et al., 2014, 2019; Lei et al., 2018), both the N1 and P2 amplitudes and the latencies of the N1 and P2 components recorded from the site Cz were selected for statistical analyses.

**Figure 3:**
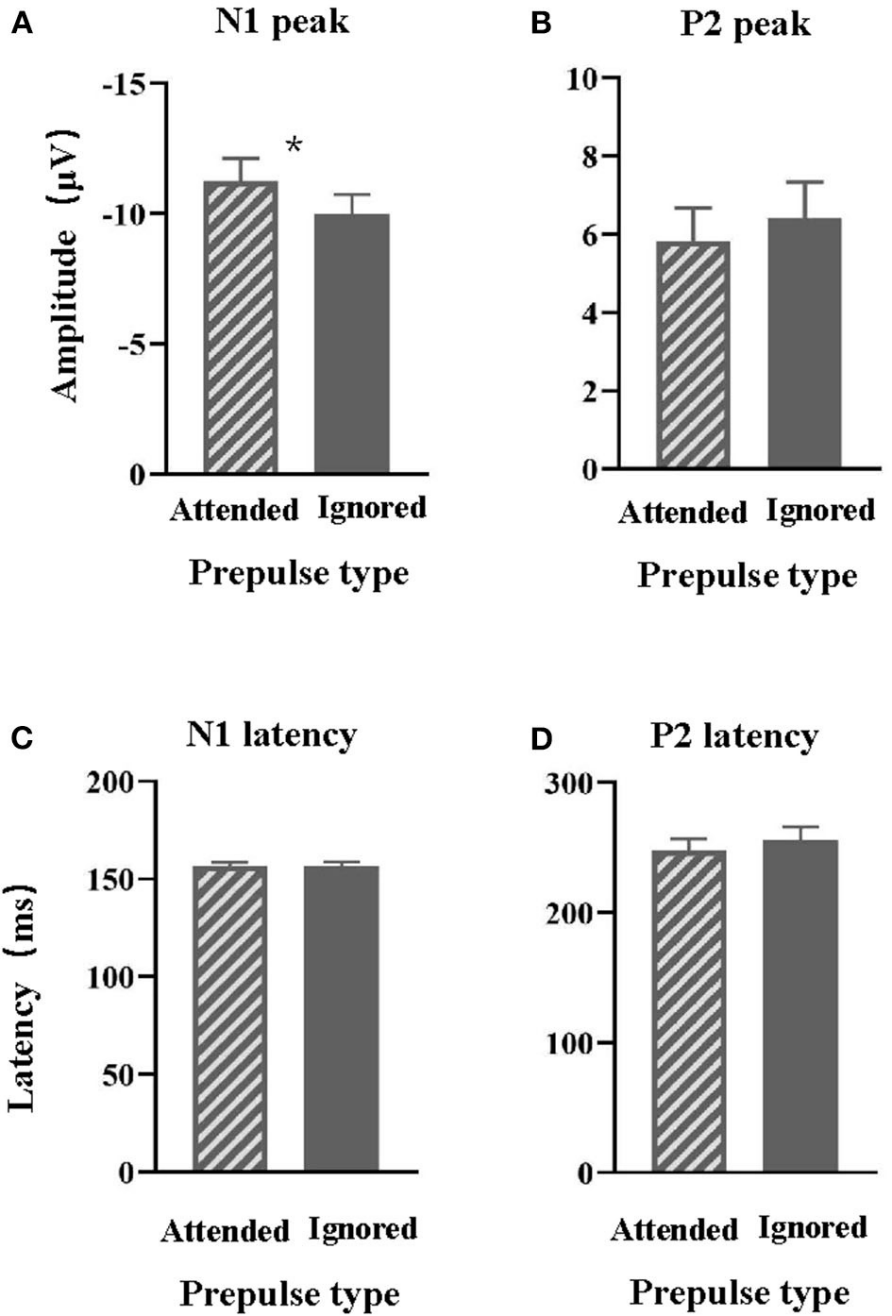
Comparisons of the group-mean N1 **(A)** and P2 **(B)** amplitudes to the prepulse stimulus recorded at the electrode site Cz between the attended prepulse condition and the ignored prepulse condition. Comparisons of the group-mean N1 **(C)** and P2 **(D)** latencies to the prepulse stimulus recorded at the electrode site Cz between the attended prepulse condition and the ignored prepulse condition. *p < 0.05.

The average values of N1 amplitudes (Figure 3A) and P2 amplitudes (Figure 3B) to the target signal across participants under each of the two conditions are displayed in Figure 3. Generally, the prepulse evoked a larger N1 wave when the signal was attended than ignored.

Separate paired *t*-tests showed that the N1 peak induced by the attended target was significantly larger than the N1 peak induced by ignored prepulse [*t* (16) = 2.54 and *p* = 0.022]. In contrast, the P2 peak induced by the attended target was not different from the P2 peak induced by ignored prepulse [*t* (16) =0.942 and *p* = 0.36].

### Latencies of ERPs to the Prepulse Stimuli

The average values of N1 latency (Figure 3C) and P2 latency (Figure 3D) to the target signal across participants, at the electrode Cz, under each of the two conditions are also displayed in Figure 3.

Separate paired *t*-tests showed that the N1 and P2 latencies were not significantly affected by the attentional modulation (*p* = 0.887 and *p* = 0.333, respectively).

### Correlation of PPIs and ERPs

**Figure 4:**
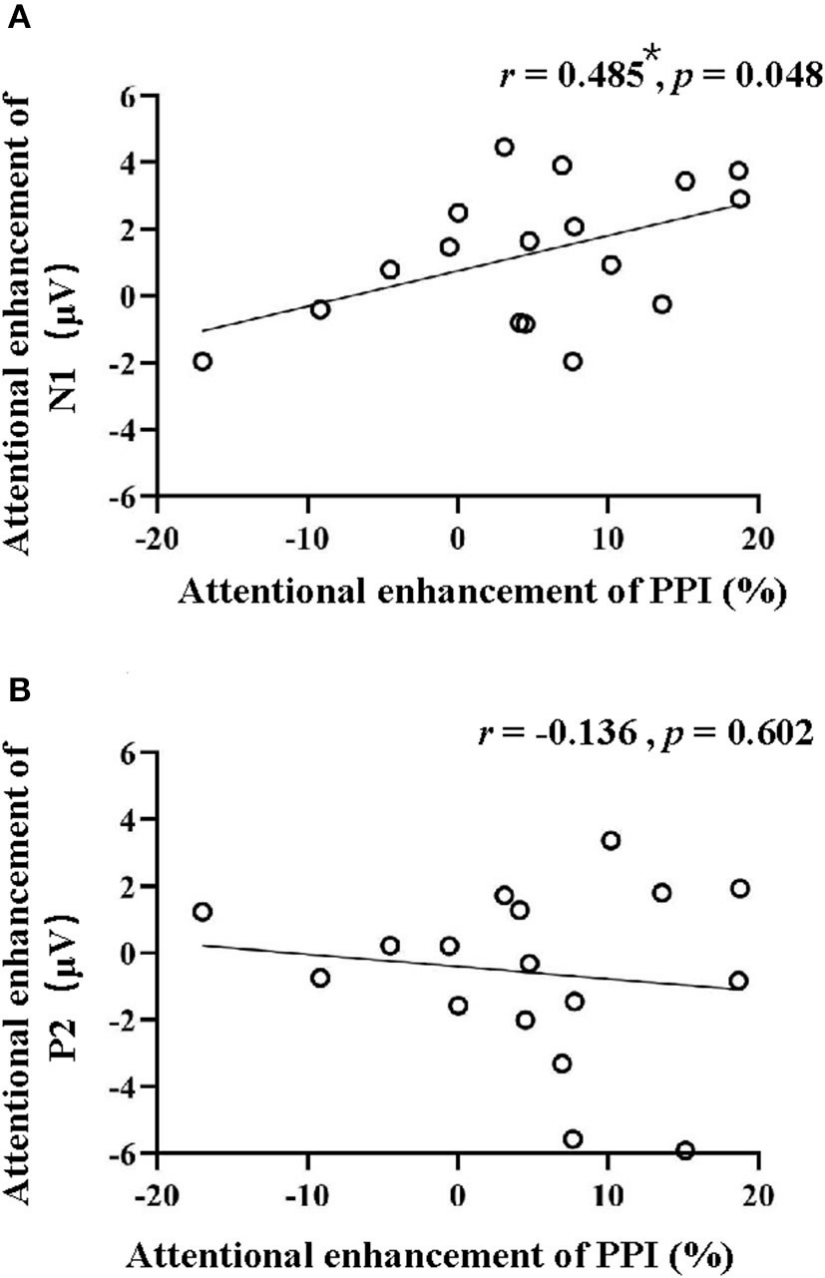
Correlations between the attentional enhancement of PPI and the attentional enhancement of N1 **(A)** and P2 **(B)**. *p < 0.05.

Figure 4 shows the correlations between the attentional enhancement of PPI and the attentional enhancement of N1 peak amplitude (Figure 4A) and P2 peak amplitude (Figure 4B). Pearson's correlation coefficients were calculated. Results showed that the attention-induced PPI enhancement significantly correlated with the attention-induced N1-component enhancement (*r* = 0.485 and *p* = 0.048, Bonferroni corrected) but not the P2-component enhancement (*r* = −0.136 and *p* = 0.602, Bonferroni corrected).

## Discussion

We have developed a novel attention-to-prepulse PPI paradigm to study the attentional modulation of PPI. We found that the discrete 150 ms narrowband noises could be used as prepulse to induce PPI with a lead interval of 270 ms, and the PPI with attended prepulse was larger than the PPI with ignored prepulse. Primarily, the early cortical representations (N1/P2 complex) of the prepulse show dissociation between the attended and ignored prepulse, where N1 evoked by the attended prepulse was larger than that evoked by the ignored prepulse. The N1-amplitude enhancement induced by attention positively correlated with the PPI enhancement induced by attention, while the P2 evoked by the attended prepulse was not significantly different from the P2 evoked by the ignored prepulse.

The attentional enhancement of PPI discovered in this study is consistent with those of previous studies (Dawson et al., 1993; Filion et al., 1993; Hazlett et al., 2003, 2007; Poje and Filion, 2021). In these studies, the continuous prepulse with a lead interval of 120 ms could elicit larger PPI when attended than when ignored. However, this attentional modulation of PPI disappeared with a discrete prepulse of 20 ms (Poje and Filion, 2021). Previous studies using longer discrete prepulse (50–150 ms) have shown that PPI can be modulated by fear conditioning and perceptual separation (Li et al., 2009; Du et al., 2011; Lei et al., 2014, 2018; Wu et al., 2019). This study found that PPI with attended long discrete prepulse (150 ms) was larger than the PPI with ignored discrete prepulse at a lead interval of 270 ms. Thus, attentional modulation of PPI exists when the prepulse is discrete (such as 150 ms narrowband noise).

In addition, PPI is modified by attentional processes depending on the lead interval used (Filion et al., 1993; Heekeren et al., 2004; Li et al., 2009; Lei et al., 2018). In Filion's PPI protocol, only with 120 ms lead interval, PPI can be enhanced when attended. Long lead intervals (100–270 ms) have been approved to be efficient in eliciting modified PPI in other studies (Du et al., 2011; Lei et al., 2014, 2018; Wu et al., 2019). This study demonstrated that the attentional modulation of PPI occurs when the lead interval is set at 270 ms.

Previous studies have also found that spatial attention to the prepulse induced by the perceptual separation between the prepulse and noise masker could induce PPI enhancement. This novel attentional modulation of the PPI paradigm showed that PPI could be enhanced by non-spatial attention. Further studies combining the non-spatially and spatially attentional modulation of PPI are needed to further investigate the complex mechanism underlying the PPI modulated by the two attention forms.

Furthermore, PPI can index automatic and attention-modulated aspects of sensorimotor gating. Automatic sensorimotor gating is typically assessed by a no-task PPI protocol (automatic PPI protocol), and attention-modulated sensorimotor gating is typically assessed through a task-based PPI protocol (attend-to-prepulse PPI protocol). In the future study, to further investigate the attentional effect on PPI, we can combine no-task PPI protocol and attend-to-prepulse PPI protocol and specify the attentional condition of participants to compare the PPI with attended prepulse, the PPI with ignored prepulse, and the PPI with unmanipulated prepulse.

The electrophysiological results of the present study showed that attended prepulse compared with the ignored prepulse could significantly enhance the early cortical representation of the target signal (the N1 component), and the N1 increase positively correlated with the PPI increase induced by attention, whereas the P2 component was not affected by the attentional modulation. This study result is consistent with a recent ERP research (Lei et al., 2018), showing that N1 and P2 components are both affected by perceptual separation, whereas only N1 increase is correlated with the perceptual separation induced-PPI increase. Zhang et al. (2019) has also found that when the target syllable is co-presented with noise masker, N1 is only affected by spatial attention, whereas P2 is affected by both perceptual separation and spatial attention, demonstrating a dissociation of N1 and P2 components between spatial attention and perceptual separation. The N1 component may be produced in the temporal auditory cortical fields (Woods et al., 1993) and is affected by spatial attention and feature-based attention (Bae and Luck, 2018). The P2 component may reveal the acoustically driven outputs from the mesencephalic reticular activating system, which reflects multiple sensory inputs (Crowley and Colrain, 2004); therefore, the P2 component has been reported to reflect higher-level cognitive processing such as discrimination (for review refer to Lightfoot, 2016). Since the N1 and P2 latencies in this study are not affected by attentional modulation, directed attention mainly enhances the processing depth of the prepulse but not the processing speed of the prepulse. This study found the dissociation of N1 and P2 components between the attended and ignored prepulse, suggesting that directed attention increased the early cortical representation (N1) of the attended prepulse.

Although there is a significant correlation between the PPI enhancement induced by attention and the N1 enhancement induced by attention, it does not indicate a causal link between the two variables. Future studies can use other neurophysiological techniques, such as TMS, to investigate the causal relationship between cortical changes (such as N1) and PPI modification. Also, previous studies have found that PPI is affected by gender (Swerdlow et al., 1993), smoking, and sleep deprivation (for review, refer to Kohl et al., 2013). Effects of gender, smoking, and sleep deprivation on PPI should be taken into account in future studies. In addition, in this study, we conducted the PPI test and EEG recording separately. In the future, with the help of engineers, we can try to combine the PPI equipment and EEG equipment to get the ERP and PPI data simultaneously. Finally, we have explored a relatively small number of participants in this study. Future studies can recruit more participants to examine whether the attentional modulation of PPI would be more effective for some subjects compared with other participants.

PPI deficiency has been documented in several psychiatric conditions, such as schizophrenia, bipolar disorder, and depression (Kohl et al., 2013). In order to distinguish among those psychiatric conditions, it is important to develop physiological and objective measures to help in the diagnosis and treatment of psychiatric disorders. Hazlett et al. (2007) have found that only the impairment of the attentional modulation of PPI, but not baseline PPI, is significantly correlated with the Brief Psychiatric Rating Scale (BPRS) total scores, positive and negative symptom factor scores of schizophrenia. Deficits of attentional modulation of PPI might be specific in schizophrenics. On the other hand, Braff et al. (2001) have found that discrete white noise prepulse, compared with continuous prepulse, was more effective in eliciting PPI deficits in schizophrenia patients. Thus, the modified attend-to-prepulse PPI paradigm, with discrete prepulse, might serve as a potential tool in the diagnosis and treatment of schizophrenia. In the future, it would be interesting to test whether schizophrenic patients show deficits in the novel attention-to-prepulse PPI paradigm compared with other psychiatric patients and the underlying neural correlates of this dysfunction.

In summary, this study developed a modified attend-to-prepulse PPI paradigm and tested whether discrete prepulse could elicit an attentional effect on PPI with a relatively long lead interval, and primarily, we investigated the neural correlates underlying this attentional modulation of PPI. The results reveal that, in the novel attend-to-prepulse PPI paradigm, when the discrete prepulse is set at 150 ms, directed attention significantly enhances both PPI and scalp ERPs to the prepulse stimulus. In addition, the early cortical representations (N1/P2 complex) of the prepulse show dissociation between the attended and ignored prepulse, and only the attentional enhancement of the N1 component is positively correlated with the attentional enhancement of PPI, indicating that the attentional modulation of PPI is more related with the primary auditory information processing.

## Data Availability Statement

The raw data supporting the conclusions of this article will be made available by the authors, without undue reservation.

## Ethics Statement

The studies involving human participants were reviewed and approved by Ethics Committee of the Laboratory of Artificial Intelligence and Cognition at Beijing International Studies University. The patients/participants provided their written informed consent to participate in this study.

## Author Contributions

ML, YD, and QM designed and carried out the experiments. ML analyzed the data and drafted the manuscript. All authors contributed to the article and approved the submitted version.

## Conflict of Interest

The authors declare that the research was conducted in the absence of any commercial or financial relationships that could be construed as a potential conflict of interest.
